# Sertraline to reduce recidivism in impulsive violent offenders (ReINVEST): a randomised double blind clinical trial

**DOI:** 10.1016/j.eclinm.2025.103668

**Published:** 2025-11-27

**Authors:** Tony Butler, Emaediong I. Akpanekpo, Lee Knight, Kristy Robledo, David Greenberg, Andrew Ellis, Stephen Allnutt, Kay Wilhelm, Alison Jones, Rodney Scott, Bianca Ton, Luke Grant, Philip Mitchell, Ross Tynan, Jocelyn Jones, Dominic Villa, Duncan Chappell, Carolynn Dixon, Alison Churchill, Val Gebski, Tony Keech, Peter W. Schofield

**Affiliations:** a University of NSW, School of Population Health, Sydney, Australia; b Hunter New England Local Health District, Newcastle, Australia; c University of Newcastle, Newcastle, Australia; d University of NSW, School of Psychiatry, Sydney, Australia; e UNSW School of Psychology, Sydney, Australia; f Justice Health and Forensic Mental Health Network, Sydney, Australia; g Sunshine Coast Health Institute, Sunshine Coast University Hospital, Birtinya, Queensland, Australia; h NHMRC Clinical Trials Centre, University of Sydney, Sydney, Australia; i Maladjiny Research Centre, Edith Cowan University, Perth, Australia; j New Chambers, Sydney, Australia; k Sydney University, Institute of Criminology; l Community Restorative Centre; m Corrective Services NSW

**Keywords:** Sertraline, Impulsivity, Violence, Recidivism, Domestic violence

## Abstract

**Background:**

Biological studies suggest serotonin modulation via selective serotonin reuptake inhibitors (SSRIs) might reduce impulsive violence, however, robust evidence in offender populations is limited. We aimed to determine whether sertraline reduces violent reoffending compared with placebo in highly impulsive men with recurrent violent offending.

**Methods:**

We conducted a double-blind, placebo-controlled, randomised clinical trial in community settings in New South Wales, Australia. Eligible participants were men aged 18 years or older with ≥2 prior violent convictions and a Barratt Impulsiveness Scale score ≥70. Following a 4-week single-blind active run-in phase with sertraline, participants were randomly assigned (1:1, minimization stratified by key factors) to receive oral sertraline (100 mg daily) or matching placebo for 12 months. The primary outcome was the first convicted violent offence within 12 months, assessed via linkage to the state Reoffending Database. The primary analysis followed the intention-to-treat principle. The trial is registered with ANZCTR (ACTRN12613000442707) and is closed to new participants.

**Findings:**

Between October 28, 2013, and July 13, 2021, 630 eligible men were randomly assigned: 319 to sertraline and 311 to placebo. By 12 months, only 204 participants remained engaged with the study. All participants were included in the primary analysis. A primary outcome event (violent offence within 12 months) occurred in 72 (22.6%) of 319 participants assigned sertraline and 70 (22.5%) of 311 assigned placebo (Relative Risk 1.00, 95% CI 0.75–1.34; p = 0.99). Serious adverse events occurred in 22 (6.9%) participants receiving sertraline and 29 (9.3%) receiving placebo.

**Interpretation:**

Sertraline did not significantly reduce the risk of violent reoffending compared with placebo. Post-hoc analyses suggested a possible selective effect on domestic violence offending.

**Funding:**

Initial funding for the ReINVEST trial was provided from an Australian National Health and Medical Research Council partnership grant No. 533559. From 2018, subsequent funding was provided by the 10.13039/501100025174NSW Department of Communities and Justice.

**Research in context**

**Evidence before this study**

We searched PubMed, EMBASE, PsycINFO, and the Cochrane Central Register of Controlled Trials from database inception to September 1, 2013, with no language restrictions. Search terms included combinations of “serotonin”, “SSRI”, “selective serotonin reuptake inhibitor”, “sertraline”, “violence”, “aggression”, “impulsivity”, “offender”, “recidivism”, and “randomized controlled trial”. We included randomized controlled trials and systematic reviews examining pharmacological interventions for reducing violence or aggression in adult offender populations. Biological studies demonstrated associations between low cerebrospinal fluid 5-hydroxyindoleacetic acid (5-HIAA) concentrations and impulsive and aggressive behaviour. Genetic research identified polymorphisms in serotonergic pathway genes associated with antisocial behaviour. However, no placebo-controlled trials had tested whether modulating serotonin through selective serotonin reuptake inhibitors (SSRIs) could reduce violent reoffending. Existing evidence also indicated that most perpetrator-focused interventions had limited evidence of efficacy and poor methodological quality, with most studies having small samples, short follow-up periods, and reliance on self-reported outcomes.

**Added value of this study**

This is the first large-scale, double-blind, placebo-controlled randomized trial examining whether an SSRI reduces violent reoffending. We enrolled 630 highly impulsive men with multiple violent convictions and ascertained convictions through administrative data linkage for up to 24 months. Participants had access to psychosocial support from the trial team. We found no evidence of difference between sertraline and placebo for violent offending at 12 months. At 24 months, domestic violence offending was lower in the sertraline group (28.2%) than in the placebo group (35.7%), a relative risk reduction of 21%.

**Implications of all the available evidence**

This trial provides inconclusive evidence about sertraline's effect on general violent offending. The reduction in domestic violence offending at 24 months suggests sertraline may have differential effects within domestic contexts. The divergent findings between general violence and domestic violence outcomes indicate that violence prevention may require targeted approaches. The high attrition rates observed demonstrate the challenges of conducting pharmacological trials in justice-involved populations. Future research should focus on identifying biological or psychological markers to optimize treatment response and developing strategies to enhance retention in this population.

## Introduction

Interpersonal violence is a leading cause of death and injury worldwide, significantly contributing to mortality and injury-related disability-adjusted life years (DALYs).^1^ The burden of violence perpetration is evident in the high rates of violent crime across many countries.^2^^,^^3^ In Australia, 63% of prisoners in 2024 were incarcerated for violent offences.^4^ Between 2014 and 2024, the number of court defendants whose principal offence was acts intended to cause injury increased by 74%. The rates of violent recidivism are similarly concerning, with 39% of individuals convicted of violent offences being reconvicted for another violent offence within a decade, while 64% are reconvicted for any type of offence.^5^ Domestic violence (DV) remains a significant issue in Australia, with an estimated 4.2 million adults experiencing partner violence since the age of 15 years.^6^ Despite various interventions targeted at reducing violent recidivism among male prisoners, these figures suggest that existing programs have had limited success in reducing violence, highlighting the need for alternative approaches. One area of focus is the role of serotonin in regulating aggression, driven by the hypothesis that modulating serotonin function could help mitigate violence.^7^

Biological and pharmacological evidence suggests a relationship between serotonergic dysfunction, aggression, and impulsivity. Reduced levels of 5-hydroxyindoleacetic acid (5-HIAA), a serotonin metabolite, have been correlated with measures of aggression and impulsivity across various populations, including violent offenders.^8^ Genetic research has identified variations in genes associated with serotonergic function that may be linked to antisocial behavior.^9^ Impulsivity, characterized by actions taken without adequate forethought or a predisposition toward rapid, unplanned reactions without consideration of consequences, has been strongly associated with criminal behaviour, including violent crime.^10^ Studies have shown that higher levels of impulsivity are associated with youth delinquency and self-reported criminal activity.^11^ This relationship is further reinforced by evidence demonstrating a negative correlation between self-control and self-reported delinquency.^12^

Efforts to mitigate impulsive violence have encountered limited success, as many existing intervention strategies do not yield sustained improvements. A recent umbrella review of perpetrator-focused interventions concluded that most studies have limited evidence of efficacy and are characterized by poor methodological quality, thereby underscoring the necessity for more robust research to evaluate the effectiveness of violence prevention strategies.^13^ Similarly, interventions designed to reduce violent recidivism and domestic violence are supported by a weak evidence base and demonstrate limited effectiveness.^14^, ^15^, ^16^ However, emerging evidence suggests a potentially effective approach. In a pilot open-label trial investigating the impact of sertraline on men with histories of violent offending who were assessed as impulsive, promising but preliminary results were observed. After three months of treatment, reductions were reported in measures of impulsivity, irritability, anger, and assaultive behavior.^7^ However, the open-label design and small sample size limited definitive conclusions regarding efficacy. Motivated by these initial findings and the need for rigorous evidence, we conducted the ReINVEST trial: a large-scale, double-blind, placebo-controlled randomized trial of sertraline among highly impulsive men in the community with prior convictions for violence to assess its effectiveness in reducing objectively measured reoffending.

## Methods

### Trial design and oversight

The ReINVEST trial was a parallel, two-arm double-blind, placebo-controlled randomized clinical trial, with a single-blind run-in phase, in community settings in New South Wales, Australia. The trial was designed to evaluate the effectiveness of sertraline in reducing reoffending rates among individuals with a history of violent offending. Prior to commencement, the study received ethical approval from multiple independent committees: UNSW Human Research Ethics Committee (HREC) (HC11390, HC17771), Aboriginal Health & Medical Research Council HREC (822/11), and Corrective Services NSW HREC (09/26,576). Additional approval was obtained from NSW Justice Health and Forensic Mental Health Network HREC (G8/14) to enable participants to continue the study medication in the event of incarceration during the trial period.

### Participants

Men aged 18 years or older with at least two prior convictions for violent offences, including but not limited to manslaughter, robbery, assault, and domestic violence, were included in the study. Eligible individuals scored 70 or higher on the Barratt Impulsiveness Scale (BIS) and were medically fit to undertake the trial.^17^ Participants were required to provide informed consent and possess the ability to communicate in English. Detailed eligibility criteria are provided in the Supplementary Appendix 1.

### Trial setting

Participants were recruited through several sources, including referral from: (1) magistrates and lawyers in local courts within the Sydney metropolitan and outer metropolitan areas, as well as the Central Coast and Hunter regions of New South Wales; (2) community corrections officers in the case of those individuals serving community sentences or on parole; (3) self-referrals from promotional materials in courts and community corrections offices and a dedicated study website. Trial recruitment was discontinued due to a lack of further funding, despite the necessity for a larger sample size to mitigate the high attrition rates typically observed in justice-involved populations. Participants received financial reimbursement to cover travel expenses and incidentals: AUD $50 for the initial screening, AUD $20 for each follow-up assessment visit, and AUD $10 for each medication collection visit.

### Trial procedures, randomization, and blinding

Following screening by a nurse to determine eligibility and a psychiatrist to determine medical fitness, the trial began with a 4-week active run-in phase, when all participants received single-blind sertraline tablets, up-titrated over three days to 100 mg, identifying those who reacted poorly to the medication or were unwilling to commit to 12 months of follow-up (Supplementary Appendix 2). A subset of participants entered the run-in phase but were not randomized for a variety of reasons, as explained below. Those deemed still eligible were then randomized to receive either sertraline or a matching placebo in a 1:1 ratio using minimization techniques. The stratification factors included age (<30 years/≥30 years), Aboriginal or Torres Strait Islander status (Yes/No), BIS score (70–90/91–120), Level of Service Inventory-Revised (LSI-R) score (14–33/34–47/absent), and study location (15 sites).

Blinding was maintained using a unique medication kit number, with participants receiving a Webster Pack containing two weeks' worth of medication upon randomization. Those assigned to placebo tablets after the run-in period were titrated down to 50 mg/day for one week and received placebo for the second week. In contrast, participants in the active arm (i.e., those continuing on sertraline) received 100 mg/day of sertraline for both weeks. The Webster Packs for both groups had no discernible differences between the active and placebo tablets. A team of mental health nurses distributed the medication kits to participants during monthly face-to-face follow-up assessments, thereby preserving the double-blind nature of the study throughout its duration.

All participants, regardless of treatment allocation, had access to a comprehensive support structure provided by the trial's clinical team throughout their participation. This included scheduled consultations, as well as additional consultations as needed, with trial psychiatrists, mental health nurses, and psychologists during follow-up contacts. Referrals to external social support services (e.g., housing, welfare, employment assistance, social work services) were also provided as appropriate.

The primary randomized treatment phase was planned for 12 months. However, provisions were made allowing participants, in consultation with the trial clinicians, the option to continue receiving the study medication (sertraline or placebo, maintaining blinding) beyond the formal 12-month endpoint if considered clinically appropriate and feasible. Throughout the trial, participants were contacted weekly to inquire about their well-being and had access to a 24-h toll-free phone number monitored by one of the trial clinicians. A medical assessment committee composed of senior clinicians managed clinical aspects of patient care. No formal stopping rules were included in the design, and safety aspects of the trial were periodically reviewed by an Independent Data Safety Monitoring Board.

### Statistical considerations

#### Outcomes

The pre-specified primary outcome was the occurrence of any convicted violent offence within 12 months post-randomization, determined through record linkage to the New South Wales Bureau of Crime Statistics and Research's Reoffending Database (ROD). In NSW, local courts typically finalise 100% of summary criminal trials and 100% of indictable matters discharged or committed for trial or sentence to the Supreme or District Court within 12 months.^18^ Violent offences were identified from the ROD according to the Australian and New Zealand Standard Offence Classification (ANZSOC) categories (2011 release): 01 (homicide and related offences); 02 (acts intended to cause injury); 03 (sexual assault and related offences); 04 (dangerous and negligent acts endangering persons); 05 (abduction, harassment, and other offences against the person); and 06 (robbery, extortion, and related offences).^19^

Pre-specified secondary outcomes included: (1) the occurrence of any convicted domestic violence (DV) offence; (2) self-reported offending, (3) any substance use (4) alcohol use and (5) changes from baseline (screening visit prior to the run-in phase) to 22-week follow-up in psychometric measures of impulsivity, anger, aggression, irritability, depression, and psychological distress. The Beck Depression Inventory score was assessed at 14 weeks as specified in the protocol. All psychometric measures were assessed using validated instruments. These instruments included the Barratt Impulsiveness Scale, Kessler-10 Psychological Distress Scale, SF-12 Health Survey (Mental and Physical Health components), Anger, Irritability and Assault Questionnaire (AIAQ), State-Trait Anger Expression Inventory (STAXI-2), EYSENCK Impulsivity Scale, and Beck Depression Inventory.

DV offences were identified via linkage to the ROD using specific law part codes designated as domestic violence-related within the database. These codes identified violent acts (e.g., assault), breaches of protective orders, and property damage occurring within a domestic context (see Supplementary Appendix 2 for full list of codes). Approximately half of the DV offences were co-coded as violent offences (i.e., from ANZSOC categories 01–06).

Adherence to the study medication was assessed weekly and defined as taking the trial medication on five or more days per week (i.e., ≥70% of prescribed doses). Participants who did not attend follow-up meetings and therefore did not receive study medication were thereafter deemed nonadherent.

### Sample size

The required sample size was determined based on the anticipated rate of first violent re-offence in the control group, estimated to be 33% within 12 months.^20^ A minimum absolute reduction of 12% with sertraline treatment was considered clinically significant and sufficiently impactful to influence clinical practice. Using these parameters, we calculated that a sample size of 460 patients (230 per group) would provide 80% power to detect this difference with an alpha level of 0.05, allowing a cross over or non-adherence rate of 3.8%. We randomized 630 participants (319 to sertraline and 311 to placebo), surpassing our initial target sample size.

### Analyses

All analyses were conducted in accordance with the intention-to-treat principle. Unadjusted generalized linear model (GLM) with a log-link function was used to estimate relative risks (RR) along with 95% confidence intervals (CI). The follow-up period began on the first day post-randomization and continued until the earliest occurrence of a proven offence or 12 months after randomization. Anticipating that some participants might be incarcerated during this follow-up period for offences committed both prior to and after randomization, the observation period was extended by the duration of custody for these cases, ensuring a full 12 months of exposure for all participants.

Pre-specified secondary analyses were conducted on participant-reported outcomes. For continuous outcomes, between-group differences in changes from screening were compared using t-tests, while categorical outcomes used chi-squared tests.

Several post-hoc analyses were conducted. First, as prespecified in the analysis plan to assess potential trial effects and generalizability, we compared outcomes among three groups: participants who met eligibility criteria and commenced the run-in period but were not randomized, those randomized to sertraline, and those randomized to placebo. To account for observed baseline differences between the randomized and non-randomized groups, these comparisons were also performed using multivariable analyses adjusting for baseline covariates. Second, since some individuals elected to continue on study medication up to 24 months post randomization and outcome data was available through administrative data linkage to the Reoffending Database beyond the pre-specified 12-month period, we conducted a post-hoc analysis examining outcomes over a 24-month follow-up period. Third, further analyses examined the primary and secondary offending outcomes at 12 and 24 months stratified by treatment adherence.

No imputation was performed for missing data. Offending outcomes ascertained through administrative record linkage was available for all participants. Analyses of participant-reported secondary outcomes were conducted with the use of a complete-case method.

Statistical analyses were conducted using R version 4.4.0 and Stata version 19.5 (StataCorp, College Station, TX). The trial has been reported according to CONSORT guidelines (see Supplementary Materials).

### Role of the funding source

The funder of the study had no role in study design, data collection, data analysis, data interpretation, or writing of the report.

## Results

### Participants

**Fig. 1:**
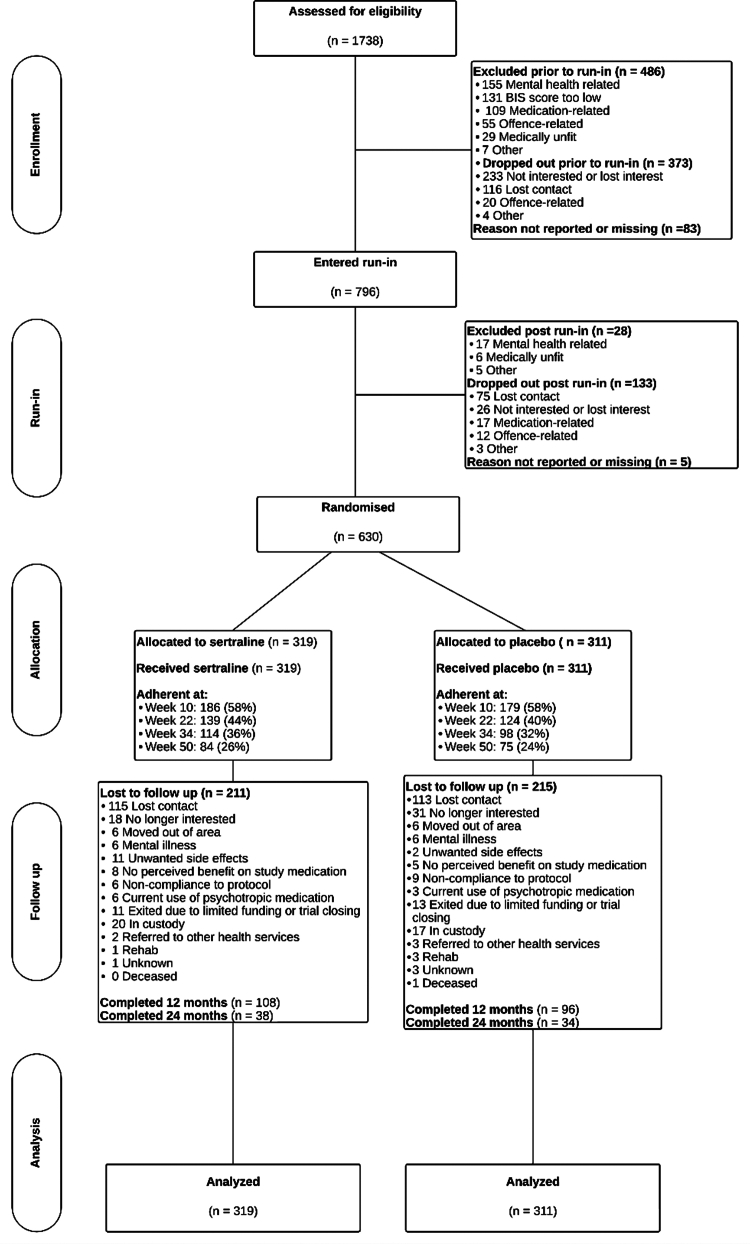
**CONSORT flow diagram of participant progress through the trial**.

Recruitment for the trial occurred from October 2013 to July 2021. A total of 1738 individuals were screened for the study (see Fig. 1). After multiple stages of screening, which included a medical assessment, 166 entered the run-in phase but were not randomized. Reasons for non-randomization after starting the run-in included exclusion (n = 28), loss of contact (n = 75), adverse events (n = 11), lack of perceived benefit (n = 6), loss of interest (n = 26), offence-related reasons (n = 12), other (n = 3), and missing (n = 5). This non-randomized run-in group served as a comparison cohort for the ascertainment of generalizability and trial participation effects. The 630 participants who completed the run-in phase were randomly assigned to receive either sertraline (n = 319) or a placebo (n = 311). The baseline demographic, criminographic, and psychometric characteristics of the study population are presented in Table 1. Baseline characteristics were balanced between the two groups, with a median age of 32 years in both groups (range: 18–66). Indigenous individuals constituted 30% of the trial participants. In the six months preceding randomization, 61% of participants reported being employed. Additionally, 22% had experienced incarceration during their adolescence.

**Table 1:** Characteristics of the participants at baseline.

Characteristics	Overall N = 630	Sertraline n = 319	Placebo n = 311
Age at randomisation (years), Median (IQR)	32 (25, 40)	32 (25, 39)	32 (25, 41)
Unknown	9	5	4
Country of Birth			
Australia	520 (83%)	256 (81%)	264 (85%)
Other	107 (17%)	62 (19%)	45 (15%)
Unknown	3	1	2
Indigenous status			
Indigenous	188 (30%)	100 (32%)	88 (28%)
Non-Indigenous	440 (70%)	217 (68%)	223 (72%)
Unknown	2	2	0
Marital status			
Single	328 (52%)	164 (52%)	164 (53%)
Partner	154 (25%)	84 (26%)	70 (23%)
Married/Defacto	99 (16%)	50 (16%)	49 (16%)
Separated	34 (5.4%)	16 (5.0%)	18 (5.8%)
Divorced	10 (1.6%)	3 (0.9%)	7 (2.3%)
Widowed	1 (0.2%)	0 (0%)	1 (0.3%)
Unknown	4	2	2
Number of children/stepchildren	1.00 (0.00, 3.00)	1.00 (0.00, 3.00)	1.00 (0.00, 3.00)
Unknown	2	1	1
Accommodation			
Renting	425 (68%)	220 (69%)	205 (66%)
Home/Family	136 (22%)	62 (19%)	74 (24%)
Unsettled	30 (4.8%)	17 (5.3%)	13 (4.2%)
Sleeping rough	7 (1.1%)	4 (1.3%)	3 (1.0%)
Other	30 (4.8%)	15 (4.7%)	15 (4.8%)
Unknown	2	1	1
Highest education level			
Never attended school	1 (0.2%)	1 (0.3%)	0 (0%)
Primary school	14 (2.2%)	6 (1.9%)	8 (2.6%)
Left school with no qualification	200 (32%)	106 (33%)	94 (31%)
School certificate	221 (35%)	115 (36%)	106 (35%)
HSC/Leaving certificate	63 (10%)	32 (10%)	31 (10%)
College/Diploma	25 (4.0%)	10 (3.2%)	15 (4.9%)
Technical or Trade qualification	87 (14%)	39 (12%)	48 (16%)
Degree/Tertiary qualification	13 (2.1%)	8 (2.5%)	5 (1.6%)
Unknown	6	2	4
Working in the past 6 months	380 (61%)	189 (59%)	191 (62%)
Unknown	5	1	4
Juvenile detention	140 (22%)	73 (23%)	67 (22%)
Unknown	5	2	3
Parent in prison when child	139 (22%)	63 (20%)	76 (25%)
Unknown	5	3	2
Number of previous offences^h^			
Any offence	9 (4, 15)	9 (4, 17)	9 (5, 15)
Violent offence	2 (1, 4)	2 (1, 4)	2 (1, 3)
Domestic violence (DV) offence	2 (0, 4)	2 (0, 4)	2 (0, 4)
Violent DV offence	1 (0, 2)	1 (0, 2)	1 (0, 2)
Barratt Impulsiveness Scale - Total Score, Median (IQR)^a^	85 (77, 92)	85 (77, 91)	85 (77, 92)
Kessler-10 - Psychological Distress Scale, Median (IQR)^b^	14 (8, 21)	14 (8, 20)	14 (9, 22)
SF-12 - Mental Health Score, Median (IQR)^c^	43 (30, 52)	43 (30, 52)	43 (31, 51)
SF-12 - Physical Health Score, Median (IQR)^c^	55 (51, 58)	55 (51, 58)	55 (51, 57)
AIAQ Total Score, Median (IQR)^d^	72 (56, 88)	71 (55, 86)	73 (56, 89)
STAXI - Anger Expression Index, Median (IQR)^e^	51 (43, 60)	50 (42, 59)	52 (43, 61)
EYSENCK – Impulsivity Score, Median (IQR)^f^	14 (11, 16)	14 (12, 16)	14 (11, 16)
Beck Depression Inventory Score, Median (IQR)^g^	9 (4, 15)	8 (4, 15)	10 (4, 16)

Percentages may not total 100 due to rounding. IQR denotes interquartile range.

a Barratt Impulsiveness Scale: Higher scores indicate greater impulsivity.

b Kessler-10 Psychological Distress Scale: Higher scores indicate greater psychological distress.

c SF-12 Mental and Physical Health components: Higher scores indicate better health status.

d AIAQ (Anger, Irritability and Assault Questionnaire) Total Score: Higher scores indicate greater anger and irritability.

e STAXI (State-Trait Anger Expression Inventory) Anger Expression Index: Higher scores indicate greater anger expression.

f EYSENCK Impulsivity Score: Higher scores indicate greater impulsivity.

g Beck Depression Inventory: Higher scores indicate more severe depressive symptoms.

h Within five years prior to randomization.

### Compliance with treatment and serious adverse events

At randomisation (Week 0), 93% of participants in the sertraline group and 91% in the placebo group were adherent (Supplementary Table S1). By Week 10 (approximately 3 months), adherence declined to 58% in both groups, declining further over time to ∼25% at 12 months.

Following the completion of the 12-month treatment phase, 178 participants (28.3% of those randomized) received the study medication at the first visit beyond 12 months (week 54). As detailed in Fig. 1 and, 38 participants (11.9%) in the sertraline group and 34 participants (11.0%) in the placebo group continued in the trial until 24 months. In terms of safety, serious adverse events (SAEs) occurred in 11 (6.6%) participants in the active run-in group, 29 (9.3%) in the placebo group, and 22 (6.9%) in the sertraline group (see Supplementary Tables S2 and S3 for details).

### Primary outcome

Over the 12-month follow-up period, 142 out of 630 participants (22.5%) experienced a primary outcome event (violent offence post-randomization) (Table 2). The median duration from the violent offence date to its finalisation by the court was 157 days (IQR [Interquartile range]: 67, 308). There was no evidence of a difference in the proportion of violent offences between the sertraline group (22.6%) and the placebo (22.5%) groups, with a relative risk (RR) of 1.00 (95% CI, 0.75, 1.34).

**Table 2:** Primary and secondary categorical outcomes.

Outcome	Sertraline (n = 319)		Placebo (n = 311)		Relative risk (95% CI)	P value
	Data available	N (%)	Data available	N (%)		
**Primary**						
Violent offending	319 (100%)	72 (22.6%)	311 (100%)	70 (22.5%)	1.00 (0.75, 1.34)	0.985
**Secondary**						
Domestic violence offending	319 (100%)	61 (19.1%)	311 (100%)	77 (24.8%)	0.77 (0.57, 1.04)	0.089
Self-reported offending^a^ at week 22^a^	279 (88%)	50 (18%)	278 (88%)	53 (19%)	0.94 (0.66, 1.33)	0.728
Substance use^a^ at week 22	144 (45%)	98 (68.1%)	132 (42%)	103 (78.0%)	0.87 (0.76, 1.01)	0.063
Alcohol use^a^ at week 22	144 (45%)	53 (36.8%)	132 (42%)	64 (48.5%)	0.76 (0.58, 1.00)	0.050

Violent offending was ascertained from Australian and New Zealand Standard Offence Classification (ANZSOC) Codes Division 01 to 06.

Domestic violence offending was ascertained from domestic violence-related law part codes.

a Self-reported outcomes.

### Secondary outcomes

The pre-specified secondary outcome (DV within 12 months post-randomization) occurred in 138 out of 630 participants (21.9%) and involved 14 of the 228 distinct law part codes for DV (see Appendix 2). The proportion of DV offences was lower in the sertraline group (19.1%) compared to the placebo group (24.8%) (RR 0.77; 95% CI, 0.57, 1.04), but this difference was not statistically significant. The median duration from the domestic violence offence date to its finalisation by the court was 97 days (IQR: 42, 213). Among participants providing data at mid-trial, no statistically significant differences were observed between the sertraline and placebo groups for self-reported offending or changes in psychometric measures (Tables 2 and 3).

**Table 3:** Participant-reported outcomes (secondary) at mid-trial.

Characteristic	Sertraline		Placebo		Mean difference in change (95% CI)	P value
	Data available (%)	Mean ± SD change from baseline	Data available (%)	Mean ± SD change from baseline		
BDI Score	175 (54.9%)	−5.97 ± 8.65	161 (51.8%)	−5.54 ± 8.44	−0.43 (−2.26, 1.41)	0.649
BIS: Total Score	147 (46.1%)	−17.66 ± 13.61	132 (42.4%)	−18.90 ± 14.03	1.24 (−2.02, 4.51)	0.455
BIS: Non-planning Impulsivity	150 (47.0%)	−5.31 ± 5.90	133 (42.8%)	−5.68 ± 6.22	0.37 (−1.05, 1.79)	0.609
BIS: Motor Impulsivity	150 (47.0%)	−7.25 ± 5.72	134 (43.1%)	−7.42 ± 5.95	0.17 (−1.20, 1.54)	0.805
BIS: Attentional Impulsivity	147 (46.1%)	−5.19 ± 5.16	134 (43.1%)	−5.81 ± 4.55	0.62 (−0.52, 1.76)	0.283
AIAQ: Total Score	134 (42.0%)	−25.13 ± 23.92	124 (39.9%)	−25.35 ± 25.81	0.22 (−5.90, 6.34)	0.944
AIAQ: Indirect Assault	142 (44.5%)	−2.68 ± 3.38	131 (42.1%)	−2.79 ± 4.04	0.11 (−0.78, 1.00)	0.807
AIAQ: Verbal Assault	137 (42.9%)	−4.88 ± 6.23	130 (41.8%)	−4.78 ± 5.82	−0.11 (−1.56, 1.35)	0.886
AIAQ: Direct Assault	139 (43.6%)	−7.01 ± 7.08	130 (41.8%)	−6.37 ± 8.10	−0.65 (−2.48, 1.19)	0.489
AIAQ: Labile Anger	141 (44.2%)	−4.69 ± 5.79	130 (41.8%)	−5.54 ± 5.49	0.85 (−0.50, 2.20)	0.216
AIAQ: Irritability	141 (44.2%)	−5.45 ± 6.83	129 (41.5%)	−5.59 ± 7.06	0.14 (−1.53, 1.81)	0.867
Kessler-10 Psychological Distress Scale	151 (47.3%)	−5.83 ± 10.59	136 (43.7%)	−6.93 ± 8.71	1.10 (−1.14, 3.34)	0.336
SF12: Mental health score	140 (43.9%)	6.35 ± 14.76	129 (41.5%)	8.51 ± 13.92	−2.16 (−5.60, 1.29)	0.219
SF12: Physical health score	140 (43.9%)	−0.05 ± 7.96	129 (41.5%)	−0.37 ± 9.98	0.32 (−1.86, 2.50)	0.771
STAXI2: Anger Expression Index	148 (46.4%)	−14.00 ± 14.57	136 (43.7%)	−16.60 ± 14.50	2.60 (−0.80, 5.99)	0.134

Change from baseline values are calculated as the mean difference (95% CI) between mid-trial (week 22) scores and scores prior to run-in.

The Beck Depression Inventory (BDI) was assessed at week 14 per protocol; all other measures were assessed at week 22.

For all scales, negative mean differences indicate improvement, except for the SF-12 Mental and Physical Health subscales, where positive values indicate improvement.

BDI, Beck Depression Inventory; BIS, Barratt Impulsiveness Scale; AIAQ, Anger, Irritability and Assault Questionnaire; STAXI, State-Trait Anger Expression Inventory; SF-12, 12-Item Short Form Health Survey; SD, Standard deviation; CI, Confidence interval.

### Post-hoc analyses

#### Analysis of outcomes at the 24-month follow-up period

At the 24-month extended follow-up, there was no evidence of a difference in violent offending between the sertraline (34.5%) and placebo (33.4%) groups (RR 1.03, 95% CI 0.83, 1.28). However, consistent with the trend observed at 12 months, domestic violence offending was lower in the sertraline group (28.2%) compared to the placebo group (35.7%) over the 24-month follow-up period (RR 0.79, 95% CI 0.63, 0.99).

#### Analysis of second-year outcomes by trial engagement

To further characterize the 24-month DV finding, an analysis of second-year outcomes was conducted, stratified by trial engagement among participants who were event-free at 12 months (Supplementary Table S4). Among participants who remained engaged beyond 12 months, the rate of DV offending in the second year was lower in the sertraline group (7.8%) than the placebo group (13.8%). In contrast, among participants who had disengaged by 12 months, the rates of DV offending were similar between the sertraline (13.7%) and placebo (14.9%) groups.

#### Analysis of outcomes by adherence to treatment

Further analyses stratified outcomes by treatment adherence over the first 12 months (Supplementary Table S5). For DV offending at 24 months, sertraline was associated with a significantly lower risk compared to placebo among participants classified as Most/Medium adherent (RR 0.70, 95% CI 0.49–0.98), but not among those classified as Least adherent (RR 0.91, 95% CI 0.67–1.24), although the p-value for heterogeneity was not statistically significant (p = 0.251). No significant differences by adherence were observed for violent offending.

#### Comparisons with the non-randomized run-in group

Baseline characteristics of the participants who entered the run-in phase but were not subsequently randomized were compared to those randomized (Supplementary Table S6). Generally, the non-randomized group had higher prior offences (within 5 years preceding randomization), higher baseline anger/irritability scores (AIAQ), lower rates of recent employment (past 6 months), and higher rates of prior juvenile detention compared to the randomized group. Other measured characteristics were broadly similar.

In unadjusted comparisons over 12 months (Table 4), the rate of violent offending was 32.9% in the non-randomized run-in group, compared to 22.5% in the placebo group (RR 0.68; 95% CI 0.51, 0.92) and 22.6% in the sertraline group (RR 0.69; 95% CI 0.51, 0.92). For domestic violence (DV) offending at 12 months, the rate was 34.2% in the non-randomized run-in group, compared to 24.8% in the placebo group (RR 0.73; 95% CI 0.54, 0.97) and 19.1% in the sertraline group (RR 0.56; 95% CI 0.41, 0.76). After adjusting for measured baseline differences, the risk ratio for 12-month violent and DV offending remained lower for sertraline vs. run-in (violent offending: RR 0.73; 95% CI 0.54, 0.99; DV offending: RR 0.55; 95% CI 0.39, 0.75) (Supplementary Table S7).

**Table 4:** Run-in group comparisons (Pre-specified) and extended follow-up (Post-hoc).

Follow-up	Outcome	Group	Events/N (%)	Unadjusted risk ratio (95% CI)	P value	Adjusted risk ratio^b^ (95% CI)	P value
12 months	Violent offending	Run-in group^a^	54/164 (32.9)	Reference	–	Reference	–
		Placebo	70/311 (22.5)	0.68 (0.51, 0.92)	0.013	0.75 (0.55, 1.02)	0.064
		Sertraline	72/319 (22.6)	0.69 (0.51, 0.92)	0.013	0.73 (0.54, 1.00)	0.047
	Domestic violence	Run-in group^a^	56/164 (34.2)	Reference	–	Reference	–
		Placebo	77/311 (24.8)	0.73 (0.54, 0.97)	0.028	0.74 (0.55, 1.00)	0.049
		Sertraline	61/319 (19.1)	0.56 (0.41, 0.76)	<0.001	0.55 (0.39, 0.75)	<0.001
24 months	Violent offending	Placebo	104/311 (33.4)	Reference	–	–	–
		Sertraline	110/319 (34.5)	1.03 (0.83, 1.28)	0.782	–	–
	Domestic violence	Placebo	111/311 (35.7)	Reference	–	–	–
		Sertraline	90/319 (28.2)	0.79 (0.63, 0.99)	0.045	–	–

CI, Confidence Interval.

a Run-in group were eligible participants who participated in the single-blind run-in phase but were not randomized.

b Adjusted risk ratios are derived from binomial regression models including covariates for employment in the past 6 months, history of juvenile detention, and prior offending within the past 5 years. Adjusted analyses were not performed for 24-month comparisons between randomized groups.

## Discussion

In this randomized, placebo-controlled trial involving men with recurrent violent offending and high baseline impulsivity, sertraline treatment did not reduce violent offending (convicted violent acts against persons) compared with placebo. In pre-specified secondary analyses, sertraline was associated with a reduction in domestic violence (DV) offending (convicted violent acts, breaches, or malicious property damage within domestic relationships), although this difference was not statistically significant. The 95% confidence intervals for both analyses reflect statistical uncertainty about the precise magnitude of effects within the prespecified analytic framework. Among participant-reported secondary outcomes, a lower proportion of participants in the sertraline group reported alcohol and substance use at 22 weeks than in the placebo group.

A key observation was the apparent divergence of the sertraline effect between outcomes for DV and general violent offending. While sertraline did not significantly reduce overall violent offending, the findings for DV offending followed a different pattern. The pre-specified 12-month analysis showed a non-significant trend favouring sertraline (RR 0.77; 95% CI 0.57–1.04). In post-hoc analyses extending follow-up to 24 months, this difference reached statistical significance (RR 0.79; 95% CI 0.63–0.99). The emergence of statistical significance for an effect size of similar magnitude in the full intention-to-treat cohort likely reflects increased statistical precision from event accumulation over the longer timeframe, evidenced by narrowing confidence intervals (0.57–1.04 to 0.63–0.99) despite nearly identical point estimates (RR 0.77 vs 0.79).

Importantly, since offending outcomes were ascertained through administrative database linkage for randomized participants regardless of continued trial participation, the 24-month analysis represents the same population as the 12-month analysis, eliminating concerns about differential follow-up bias. The interpretation of this finding is further informed by the continuation of blinded treatment beyond 12 months, which may have contributed to the overall effect size. Relatedly, an analysis by adherence showed a statistically significant reduction in 24-month DV offending among participants in the medium/most adherent group, an effect not observed among those with least adherence.

The apparent specificity of sertraline's potential effect on DV offending, in contrast to general violent offending, may arise from underlying differences in the typical drivers of these offending behaviors. Direct mechanisms through which serotonin modulation might specifically impact DV are not yet fully elucidated in the literature; however, DV is often conceptualized as being characterized by heightened emotional reactivity,^21^ which may be less consistently prominent in other forms of violent offending. This conceptual distinction could suggest a plausible pathway whereby sertraline's known serotonergic actions might preferentially attenuate the emotionally reactive aggression often implicated in DV incidents. Such a targeted effect on DV could contribute to the divergence in outcomes observed in this trial.

Supporting the idea that affective states are relevant to understanding different forms of violence, the broader literature indicates associations between various negative affect profiles and intimate partner violence (IPV) perpetration, as well as links between depression and general violent crime. IPV perpetration has been associated with psychological distress symptoms.^21^ Furthermore, depression specifically has been identified as a risk factor for IPV,^22^ with men being over-represented among IPV perpetrators,^23^ and their depression sometimes linked to more serious physical violence.^24^ Similarly, some population-based studies have reported associations between depression and an increased risk of non-specific violent crime.^25^ This trial excluded individuals with a diagnosis of major depression at baseline, which complicates direct comparisons of the role of more severe depressive syndromes in violence between our study population and findings from studies that may include the full spectrum of depression severity. However, it remains clinically plausible that sertraline's effects could extend to a broader profile of negative affect.^26^^,^^27^ Baseline assessments revealed that constructs such as general psychological distress (K10), anger and irritability (AIAQ) were present across a range of severities in our sample.

The lack of a clear separation between sertraline and placebo in the primary analyses likely reflects the trial's design, in which both groups received significant psychosocial support, akin to a multicomponent intervention, from a team of experienced mental health nurses and psychologists. This support plausibly reduced offending across both groups by addressing criminogenic factors.^28^ Meta-analytic evidence indicates that such interventions can independently reduce recidivism, potentially diminishing the incremental drug effects detectable in this study.^15^ Therefore, the effect size observed in this trial represents the incremental benefit of sertraline when added to intensive psychosocial support; the absolute magnitude of sertraline's effect in settings lacking such comprehensive support remains undetermined by these data.

As pre-specified to provide context on potential trial participation effects and generalizability, comparisons were made between outcomes in the randomized participants and those eligible individuals who entered the run-in phase but were not subsequently randomized. In unadjusted analyses, the sertraline and placebo groups showed lower 12-month relative risk than this non-randomized group. Importantly, the lower risk of offending for both sertraline and placebo compared to the run-in group generally persisted after adjusting for the baseline differences observed between the groups. However, the high probability of residual confounding from unmeasured factors and selection biases in comparing randomized versus non-randomized cohorts precludes drawing firm conclusions from these comparisons regarding trial participation effects or generalizability.

Strengths of the study include the randomized placebo-controlled design resulting in unbiased comparisons of offending outcomes and providing opportunity for the collection of a wealth of behavioural and neuropsychiatric data. Additionally, opportunities were provided for the ‘wrap around supports’ provided to study participants by the clinical team, such as helping to navigate mental health, welfare, and justice systems. Outcome ascertainment for official convictions via record linkage to the Reoffending Database provided comprehensive and objective data for participants within New South Wales. This method ensured complete outcome data collection even when participants discontinued study procedures, thereby reducing potential bias in the ascertainment of conviction events within the state. No serious adverse effects associated with the study medication were identified during the trial.

The limitations of this study include: (i) the inability for many participants to adhere to treatment; (ii) consistent and uniform follow-up difficulties; (iii) challenges in navigating criminal justice systems in recruitment and outcome assessment and (iv) the substantial participant attrition, (>50% of participants lost to follow-up at 6-months). Under the intention-to-treat principle, the non-adherence (∼75% at 12 months) would dilute the estimate of the treatment effect, resulting in reduced statistical power as the study was designed based on a non-adherence rate of 3.8%. Adherence was assessed by combining two factors: firstly, whether the participant had attended a visit and therefore had obtained another 30 days of medication, and secondly by self-reported pill counts. Self-reported pill counts are likely to overestimate the true exposure and would further attenuate the treatment effect. High participant attrition resulted in a substantial rate of missing data which raises doubt on the reliability of analyses involving psychometric measures. Studies involving offender populations frequently experience challenges with participant retention due to intersecting factors that compound to create complex barriers, highlighting the need for innovative approaches to increase engagement.^29^ According to a behavioural nudges sub-study, participants who got nudges via text message had a 53% higher chance of completing the trial at the prespecified endpoint or engaging meaningfully (at least 10 post-randomization contacts).^30^ However, as this was a clinical trial, and not an established program within the justice system, it did not have access to the full support of the justice infrastructure which may have reduced such attrition.

Further research is needed to explore whether sertraline preferentially attenuates DV in individuals characterized by such broader negative affective states or pronounced emotional reactivity, and to delineate the specific mechanisms through which serotonergic interventions might differentially affect DV versus general violence in highly impulsive, violent offender populations. An analysis of whether reductions in alcohol or other substance use mediate or modify the effect of sertraline on domestic-violence outcomes should be the subject of future research. Future research should focus on identifying offender subgroups most responsive to SSRI treatment, exploring flexible dosing regimens and alternative SSRIs, and investigating genetic polymorphisms in serotonin-related pathways that may affect treatment response. Future research should also investigate the feasibility and effectiveness of long-acting injectable SSRI formulations, should they become available and suitable for this population, to potentially address adherence challenges. Combination therapies that target multiple neurobiological pathways involved in impulsiveness and aggression should be explored. Finally, longer-term follow-up studies are needed to assess whether there are any sustained effects of SSRI treatment on violent behaviour.

In conclusion, this trial does not definitively establish whether sertraline reduces violent offending in men with recurrent violent behaviour and high baseline impulsivity. The provision of significant psychosocial support in both trial arms, implemented to encourage retention and fulfill our ethical duty of care to participants, may have contributed to a reduction in overall reoffending risk. This likely increased the threshold for detecting additional treatment effects, meaning that any observed differences primarily reflect the incremental effect of sertraline within a context of high psychosocial support. Post-hoc analyses suggested a possible selective reduction in domestic violence (DV) offending over 24 months. Future studies should be adequately powered, utilizing contemporary offence rates, and ideally conducted within justice system frameworks to determine whether pharmacological interventions provide context-specific benefits when combined with targeted psychosocial support. These studies should potentially focus on subgroups identified as more likely to respond.

## Contributors

TB and PWS: Conceptualisation, methodology, project administration, resources, supervision, investigation, writing—original draft, writing—review & editing, validation, and funding acquisition. EIA: Data curation, formal analysis, validation, visualisation, writing—original draft, and writing—review & editing. KR: Investigation, methodology, formal analysis, validation, and writing—review & editing. LK: Investigation, supervision, and writing—review & editing. VG and TK: Methodology, supervision, validation, and writing—review & editing. DG, AE, SA, KW, AJ, RS, BT, LG, PM, RT, JJ, DV, DC, CD, and AC: Investigation and writing—review & editing. EIA and KR accessed and verified the data. DC could not be reached to approve the final version of this manuscript.

## Data sharing statement

The individual participant data generated and analysed during the current study are not publicly available because of specifications in participant consent forms regarding the sensitive nature of the data. De-identified data may be requested from the corresponding author, subject to relevant approvals.

## Declaration of interests

AE reports receiving payment for providing expert testimony in criminal courts and serves in unpaid roles on committees for the Royal Australian and New Zealand College of Psychiatrists and as a board member for the International College of Neuroethics and Neuroscience. KR reports her salary is supported by an NHMRC grant (#2033081). PM reports receiving support from an NHMRC Investigator Grant. All other authors declare no competing interests.

## Appendix A Supplementary data

Supplementary Material

Protocol
