# Supplementary material for: Sertraline to reduce recidivism in impulsive violent offenders (ReINVEST): a randomised double blind clinical trial
Source: eClinicalMedicine. 2025 Nov 27;90:103668. doi: 10.1016/j.eclinm.2025.103668 (PMC12702300; doi:10.1016/j.eclinm.2025.103668)
Supplement: Supplementary Material [file mmc1.docx]

**Sertraline to reduce recidivism in Impulsive Violent offenders (ReINVEST): A Randomised Double Blind Clinical Trial**

**Supplementary Appendix 1. Eligibility criteria of participants**


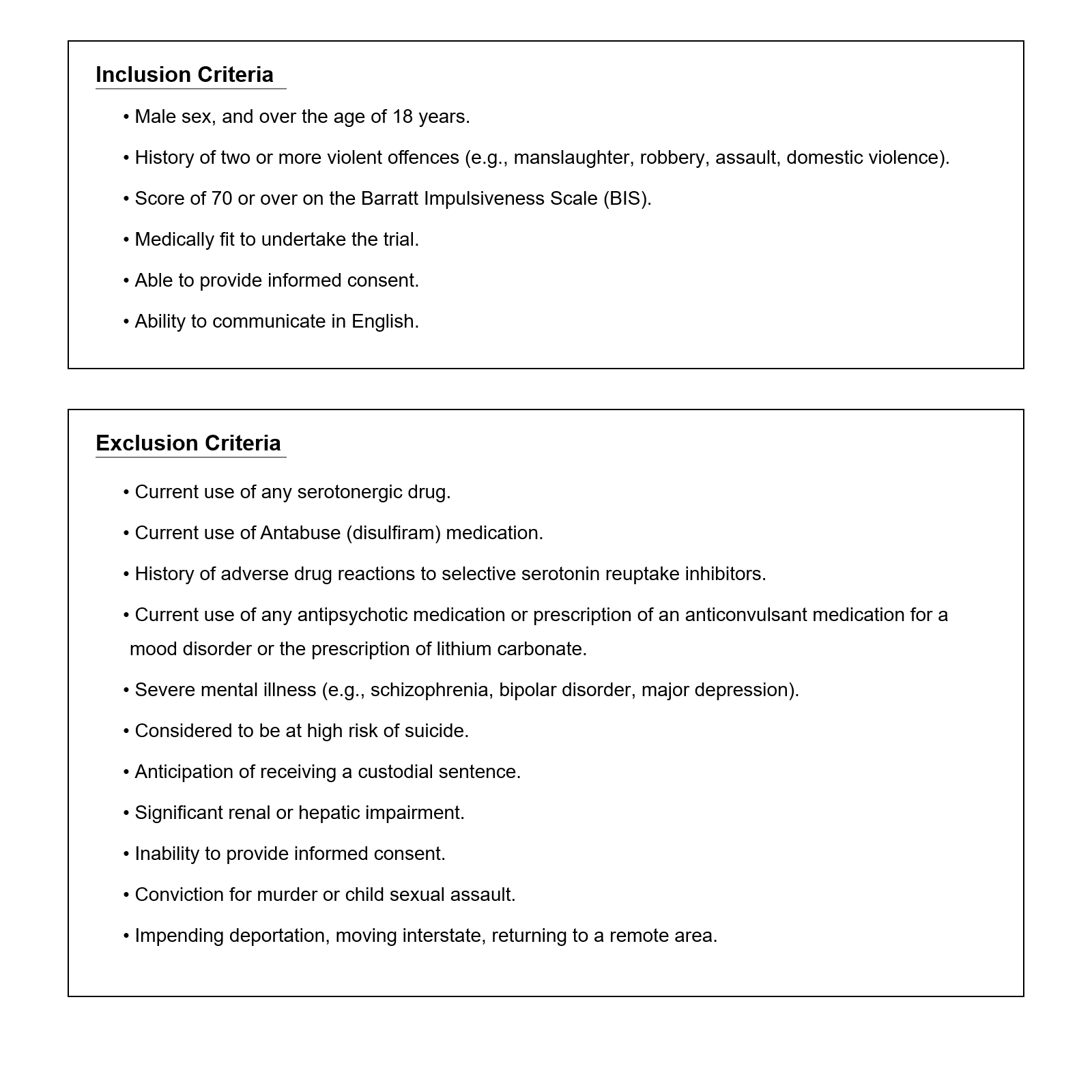


**Supplementary Appendix 2. Statistical Analysis Plan**

1. **Administrative information**
   1. **Trial name and acronym**

Reducing impulsivity in repeat violent offenders using a selective serotonin reuptake inhibitor (sertraline): the REINVEST study.

- 1. **Trial registration number**

This trial is registered at ANZCTR: ACTRN12613000442707. (<https://www.anzctr.org.au/Trial/Registration/TrialReview.aspx?id=364066>)

- 1. **Version history**

| **Protocol version** | **SAP version** | **Date** | **Changes made** | **Authors** |
| --- | --- | --- | --- | --- |
| 14047-002 | 0.1 | December 2021 | Draft document | Kristy Robledo |
| 14047-002 | 1.0 | 01 March 2022 | Incorporate recommendations from the team | Kristy Robledo |
| 14047-002 | 1.1 | 22 March 2022 | Incorporate recommendations Prof Ian Marschner for the Primary analysis wording. | Kristy Robledo |

- 1. **Contributors to the statistical analysis plan**

This statistical analysis plan has been prepared based on Australian Clinical Trials Alliance templates (<https://clinicaltrialsalliance.org.au/resource/statistical-analysis-plan/>) and JAMA guidelines (Gamble et al 2017).

| **Name** | **Affiliation** | **Role on study** | **SAP contribution** |
| --- | --- | --- | --- |
| Dr Kristy Robledo | NHMRC Clinical trials Centre, Sydney University | Trial Statistician | Draft document |
| Prof Val Gebski | NHMRC Clinical trials Centre, Sydney University | Senior statistician | Review document |
| Prof Tony Butler | School of Public Health and Community Medicine, University of new South Wales | Principal Investigator of REINVEST trial | Review document |

| Prof Peter W Schofield | School of Medicine and Public Health, University of Newcastle | Investigator of REINVEST trial | Review document |
| --- | --- | --- | --- |
| Prof Tony Keech | NHMRC Clinical trials Centre, Sydney University | CTC Clinical lead | Review document |

- 1. **Approvals**

The undersigned have reviewed this plan and approve it as final. They find it to be consistent with the requirements of the protocol as it applies to their respective areas. They also find it to be compliant with ICH-E9 principles and, in particular, confirm that this analysis plan was developed in a completely blinded manner (i.e. without knowledge of the effect of the intervention(s) being assessed).

| **Trial Statistician**  Dr Kristy Robledo | Kristy_Robl Digitally signed by  Kristy_Robledo  edo Date: 2022.03.22  14:21:15 +11'00' |
| --- | --- |
| **Head of Statistics, CTC**  Prof Ian Marschner | Ian Digitally signed by Ian Marschner  Marschner Date: 2022.03.25  10:20:33 +11'00' |
| **Chief Investigator/Clinical Lead**  Prof Tony Butler | Date: 4 March 2022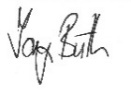 |

- 1. **Supplementary documentation**

The trial master file is located on the University of New South Wales (UNSW) servers.

This analysis plan is following the NHMRC CTC Statistical SOPs (standard operating procedures). The validation plan for REINVEST is located here:

**Y:\Statistics\1 STUDIES\Other\REINVEST\8.6 Analysis Validation**

1. **Study Synopsis**
   1. **Background and rationale**

Violence is a leading cause of death and injury worldwide, and a large percentage of prison inmates have histories of violent offending. Numerous studies attest to a strong association between poor impulse control (impulsivity) and violent crime. Impulsivity has been shown to be associated with reduced tone in neural pathways within the brain for which serotonin is the principal neurotransmitter. In a number of studies of impulsive-aggressive individuals, the administration of selective serotonin reuptake inhibitors (SSRIs) has been associated with reduced aggression, impulsivity and depression. To date, there has been no systematic study of the potential benefits of SSRIs for incarcerated impulsive violent offenders who are at high risk of repeat offending. This trial will examine the effectiveness of treatment with an SSRI in impulsive violent offenders on reoffending using a double blind RCT design.

Sertraline is an anti-depressant of the selective serotonin reuptake inhibitor (SSRI) class. It is primarily used to treat major depression in adults as well as obsessive-compulsive, panic, and social anxiety disorders in both adults and children. According to the Australian Statistics on Medicines 2007 (latest available data), SSRIs were the most commonly prescribed anti-depressant in Australia with over 8.5 million scripts issued in that year. Among SSRIs, sertraline was the most commonly prescribed anti-depressant.

- 1. **Objectives**
     1. **Primary objective**

1. Evaluate the effectiveness of SSRI (sertraline) in reducing reoffending in impulsive, repeat- violent offenders.
   - 1. Secondary objectives
2. Examine the effectiveness of SSRI (sertraline) in improving behavioural measures (impulsivity, anger, aggression, irritability, quality of life, social functioning, substance use and alcohol consumption, self-reported offending, depression and psychological distress) in impulsive, repeat-violent offenders.
3. Investigate whether polymorphisms in the serotonin transporter gene 5-HTTLPR affect the efficacy of SSRI in reducing impulsivity in impulsive, repeat-violent offenders.
   1. **Trial design**

Placebo controlled, double blind, randomised (1:1), parallel arm superiority trial with an active run in.

- 1. **Outcomes**
     1. **Primary outcome**

The primary outcome is any instance of a convicted violent offence in the 12 months post randomisation, obtained from the NSW Bureau of Crime Statistics and Research’s Reoffending Database (BOCSAR’s RoD). This includes the following Australian and New Zealand Standard Offence Classification (ANZSOC) categories:

- 01 (homicide and related offences);
- 02 (acts intended to cause injury);
- 03 (sexual assault and related offences);
- 04 (dangerous and negligent acts endangering persons);
- 05 (abduction, harassment and other offences against the person) and
- 06 (robbery, extortion and related offences).

Police records will also be checked to identify any events that may have been missed due to a time- lag prior to appearing in the BOCSAR RoD database.

We will classify patients into three mutually exclusive groups:

1. BOCSAR convicted offence within 12 months of randomisation
2. Possible conviction (obtained from charge report, with outcome pending)
3. No convicted offence*

*these are men who have no BOCSAR data and have reached 2 years post randomisation, or men who have no BOCSAR data and no pending charges after 12 months of follow-up.

The primary analysis will consider men with a conviction and a possible conviction as a primary outcome event (1+2), compared to men with no convicted offence (3).

- - 1. **Secondary outcomes**

The secondary outcomes are:

- changes at 22 weeks from screening (prior to run-in) in measures of
  - impulsivity, (BIS total score, plus subscales: motor impulsivity, attentional impulsivity and non-planning impulsivity)
  - anger (STAXI2 anger expression index),
  - aggression (AIAQ direct assault, verbal assault, indirect assault and labile anger scores),
  - irritability (AIAQ irritability score),
  - quality of life (SF12: physical and mental scores),
  - social functioning (DUKE support scale score), and
  - psychological distress (Kessler Psychological Distress Scale (K10) score)
- changes at 14 weeks from screening in depression (BDI score)
- self-reported offending at 22 weeks
- substance use at 22 weeks
  - any illegal substances (Y/N)
  - any substance use of:
    - alcohol,
    - tobacco,
    - cannabis,
    - ice/MDMA/amphetamines,
    - other substances
- alcohol consumption at 22 weeks
  - number of standard drinks per week
  - drinking categories (light, medium, heavy)
- domestic violence (DV) offence within 12 months (BOCSAR)

Another secondary outcome will be determined by any significant differences in impulsivity and depression between those with s/s versus any l/l or l/s 5-HTTLPR polymorphisms in participants given SSRI. This is outside the scope of this analysis plan.

- - 1. **Safety endpoints**

The primary safety endpoints are:

- death by suicide,
- drug overdose or
- development of serotonin toxicity.

We will also summarise all-cause mortality and SAE by treatment.

- 1. **Intervention**
     1. **Study drug**

The SSRI used in this trial is sertraline hydrochloride (SETRONA in Australia). Sertraline is primarily used to treat major depression in adults but is also indicated for obsessive compulsive disorder, premenstrual dysphoric disorder, panic disorder and social anxiety in both adults and children[.34](https://bmjopen.bmj.com/content/11/9/e044656.long#ref-34)

The trial medication comes in tablet form in a blister-pack. During dosage titration after the active run-in period, medication (active and placebo) is provided in an identical Webster Pack.

- - 1. **Placebo**

Placebo sertraline contains the inactive ingredients of SETRONA (microcrystalline cellulose, calcium hydrogen phosphate, sodium starch glycollate, hydroxypropyl cellulose, magnesium stearate, white Opadry, hypromellose, titanium dioxide, macrogol 400, purified talc). Placebo tablets are identical in shape, colour and delivery to SETRONA. The dosing is the same as the active and both tablets are taken orally.

- 1. **Randomisation and blinding**

After a four week active run in phase, eligible participants are randomised using Flexetrials, an online randomisation system. This is performed by minimisation, and stratified by age (<30 years vs ≥30 years), Aboriginal or Torres Strait islander (Yes vs No), BISII score (70-90 vs 91-120), LSIR score (14-33 vs 34-47 vs absent) and study location.

- 1. **Sample size**

The expected rate of first violent or impulsive reoffence in the control group is expected to be approximately 33% at 12 months, and a minimum of 12% reduction in this rate using sertraline would be considered sufficient to be clinically meaningful and have an impact on practice in this area. A sample size of 460 patients (230 in each group) would provide 80% power with 95% confidence to detect this difference. This sample size also allows for a cross-over/non-compliance rate of approximately 3.8%. While these estimates are conservative, if the control rate was 37%, the study would still have 80% power to detect a 12% absolute reduction. Reoffending rates will be monitored to ensure that the sample size will provide sufficient power to detect meaningful differences. This also includes an assessment of the non-compliance and cross-over rates. It is

important to consider the attrition rate; we estimate that at least 1800 men will need to be assessed at the selection phase to achieve the sample size required for the trial.

1. **Statistical principles**
   1. **General principles**

This is a superiority trial, and all analyses will be conducted according to the intention to treat principle^[[1]](#footnote-1)^. P-values less than 0.05 will be considered statistically significant, and point estimates will be given for treatment comparisons, with 95% confidence intervals (CI).

Analyses will be conducted in SAS version 9.4 and R.

- 1. **Interim analyses**

There have been no interim analyses conducted in this study.

- 1. **Timing of final analysis**

The last participant was randomised on 24^th^ September 2021, and will reach 12 months of follow-up 24^th^ September 2022. The BOCSAR ROD database will be requested at 31^st^ September 2022 and the final report will be based upon that version of the BOCSAR database.^[[2]](#footnote-2)^

- 1. **Multiplicity**

P-values will be given unadjusted for multiple comparisons.

- 1. **Missing data**

There are no plans to impute data for any analyses.

- 1. **Analysis populations**

The primary analysis will be performed on all participants who complete the randomisation visit[^1^](#_bookmark30) that are matched in the BOSCAR database, which is anticipated to be 100% of patients given previous matching. Efficacy analyses of primary and secondary outcomes will be performed using intention to treat principles.

Safety data will be reported in all participants that took study medication, which includes those men who were in the active runin and not randomised.

- 1. **Adjudication of outcomes**

For men who were randomised before September 2020, it is anticipated that all data will be present within the BOCSAR database , except in some cases of extended custody.

Men who were randomised after September 2020 and have no convicted primary outcome offence within 12 months of randomisation within the BOCSAR database, will be adjudicated for any possible charges that would meet the definition of the primary outcome offences. They will also have adjudication for any offences that could be classified as domestic violence (secondary outcome). This will be performed blinded to treatment.

1. **Analyses**
   1. **Ineligible patients**

Any participant found to be ineligible after randomisation will be summarised in the consort diagram.

- 1. **Consort diagram**

The consort diagram will include :

- Total number of screened participants
- Total number of registered participants who enter the active runin
  - Reasons participants were not registered
- Total randomised participants (by treatment)
  - Reasons participants were not randomised
- Total participants matched in BOCSAR database for primary outcome (by treatment)
  1. **Baseline characteristics**

The following participant characteristics measured at screening (or baseline) will be summarised by treatment. There will be no statistical comparisons for randomised treatment for baseline characteristics.

- Patient demographics (Age, ethnicity, marital status, children, education, employment, accommodation)
- BOCSAR data summary prior to randomisation (Previous number of offences in BOSCAR, previous number of offences meeting the primary outcome in BOSCAR, previous DV offences)
- Previous drug use

The following assessments will also be summarised by treatment at baseline.

**Supplementary Appendix Table 1: Details of Assessments performed at baseline**

| **Assessment / Procedure** | **Details** |
| --- | --- |
| Barratt Impulsiveness Scale (BIS-11)^31^ | Assess trait impulsivity |
| State Trait Anger Expression Inventory (STAXI- 2)38 | Records subjective levels of anger in different situations |
| Anger Irritability and Aggression Questionnaire^32^ (AIAQ) | Records subjective levels of anger and aggression in recent weeks. |
| Quality of Life Measures (SF-12)^35^ | Provides a validated index of subjectively perceived quality of life |
| Duke Social Support Scale^39^ | Provides a validated index of the degree of social support available to the participant |
| MINI-6 (PTSD, GAD, social phobia, ASPD, OCD, panic disorder only)^40^ | Provides an index of levels of anxiety, and personality disorder over a defined interval of time prior to the interview. |
| Personality (IPDE)^41^ | Impulsive personality disorder, dissocial personality disorder, borderline personality disorder |
| Kessler Psychological Distress Scale (K-10)^34^ | 10-item questionnaire intended to yield a global measure of distress based on questions about anxiety and depressive symptoms in past 4 weeks. |
| Alcohol Use Disorders Identification Test^42^ | Measures alcohol consumption 12 months before. Indicates safe, harmful, and hazardous alcohol use. |
| Beck Depression Inventory-II (BDI-II)^43^ | Inquires about symptoms over the preceding week indicative of a mood disorder |
| SNIFFIN sticks Identification Test (16 item version) ^20^. | This will be used to examine for anosmia (loss of sense of smell), a possible proxy for prefrontal cortex damage. |
| Eysenck Impulsivity Questionnaire (EIQ)^44^ | Self-report questionnaire on impulsive personality. |
| Wechsler Individual Achievement Test (WIAT- 11) | Provides a measure of reading ability |

- 1. **Adherence to study treatment**

Adherence to study treatment will be summarised, but as per intention to treat principles, all participants will be included in analyses. Each month participants are asked “How many days per week, on average, did you actually ingest your study medication?”. Adherent to treatment is defined as taking the trial medication for 5 in 7 days or more, i.e. more than 70% of medication. This is self reported data obtained at each monthly visit, when the next medication is dispensed. After one visit is missed, participants can be assumed to non-adherent to trial medication (as they only receive enough medication for one month at a time). The proportion of patients adherent to medication each month will be summarised by treatment.

- 1. **Withdrawal and lost to follow-up**

Participants who have missed more than three study visits in a row (therefore missed two or more months of study treatment) will be considered to be lost to follow-up. Participants that are known to have withdrawn from the trial will also be summarised by treatment, with reasons for withdrawal.

- 1. **Analysis of the primary outcome**

The primary outcome data will be obtained from the BOSCAR RoD database. Matching of clinical trial participants is performed by BOCSAR by two identifiers (MIN and CRN). If these are unavailable for participants, full names and date of birth can be used as an alternative.

If any participants are not matched within the BOCSAR database, their baseline characteristics will be summarised compared to those that were matched in BOCSAR.

The primary analysis will be composed of the participants who are able to be matched in the BOCSAR database, which we anticipate will be all trial participants. For those patients with less than 2 years of follow-up with BOCSAR and no known primary outcome offences, blinded adjudication will take place to ensure that no charges are currently pending within the court system (see section [3.7:](#_bookmark29) [Adjudication of outcomes](#_bookmark29)).^[[3]](#footnote-3)^

Participants will then be placed in one of the following mutually exclusive groups for their status at 12 months after randomisation:

- ‘convicted offence’ BOCSAR convicted offence
- ‘possible offence’ Possible conviction, pending court case (from charge report)
- ‘no offence’ No convicted offence or pending offences

Time is based upon the date of the offence, not the date of the conviction or charge date. If the participant is taken into custody within this 12 month period, then the 12 month period will be extended to account for this time in custody. Eg. if a participant is in custody for 3 months, then the total time for this participant to have a primary outcome offence will be extended to 15 months.

- - 1. **Primary analysis**

The primary analysis will be a binary regression with the use of a log link, for the outcome of convicted or possible offence versus no offence. This will enable relative risks (RR) and associated 95% CI to be obtained. The p-value for SSRI treatment from this model will test the effect of treatment. The total exposure time will be included in this analysis to account for any patients who have not reached 12 months due to time in custody.

- - 1. **Additional analyses**

An adjusted analysis will be performed for the primary outcome, adjusting for the number of previous offences before randomisation that meet the primary outcome definition.

A multinomial logistic regression (logistic regression with a generalised logit link) will be performed for the three category outcome (offence , possible offence or no offence) in order to obtain odds ratios and 95% CI for the effect of treatment, using ‘no offence’ as the reference outcome. Therefore an effect of SSRI treatment will be obtained for those with a ‘convicted offence’ compared to ‘no offence’ and also an effect of SSRI treatment will be obtained for those with a ‘possible offence’ compared to ‘no offence’.

The following additional tables will be given by treatment and tested with a chi-squared test:

- Multiple primary outcome offences (categorised as: 0, 1, 2, 3 or more*) within 12 months
- Any convicted offences within 12 months (any versus none)

*categorises may be collapsed further if the cell counts are <5

Offences by treatment and by Australian Standard Offence Classification (ASOC) will also be summarised graphically.

- 1. **Analyses of the secondary outcomes**

Analyses of the secondary outcomes will be restricted to the data completed at that timepoint. No data imputation will be performed.

- - 1. **Change in PROs from screening**

The following PROs are measured at 22 weeks:

- impulsivity,
  - BIS total score,
  - motor impulsivity,
  - attentional impulsivity
  - non-planning impulsivity
- anger
  - STAXI2 anger expression index
- aggression
  - AIAQ direct assault score
- irritability
  - AIAQ irritability score
- quality of life
  - SF12-physical
  - SF12-mental
- social functioning
  - DUKE support scale score
- psychological distress
  - Kessler Psychological Distress Scale (K10) score

And at 14 weeks :

- depression
  - BDI score

The changes in PROs are changes at 22 weeks (or 14 weeks for BDI) from screening, prior to run-in. The changes over time will be summarised graphically, for all patients and also for patients who only complete until the 22 week timepoint.

To compare the change from screening, independent 2-sample t-tests will be performed to compare treatment arms (as appropriate). Mean changes from baseline and 95% CI will be calculated.

- - 1. **Self reported offending**

Self reported offending (Y/N) will be compared between treatment arms at 22 weeks with a chi- squared test.

- - 1. **Substance use**

The use of the following types of substances will be compared at 22 weeks between treatment arms:

- Any substances
- Any illegal substances
- Alcohol
- Tobacco
- cannabis
- ice/MDMA/amphetamines
- other substances (including heroin, opiates, cocaine, etc)

Types of substance use will be compared between treatment arms with a chi-squared test. If appropriate, amounts of substance use may also be compared.

- - 1. **Alcohol consumption**

The number of standard drinks of alcohol consumed per week by those that reported use of alcohol at 22 weeks will be explored by treatment. The number of standard drinks per week will also be summarised categorically as:

- None
- Light (3 or less drinks per week)
- Moderate (4-14 drinks per week)
- Heavy (>14 drinks per week)
  - 1. **Domestic violence offences**

In a similar fashion to the primary outcome, participants will be placed in one of the following three mutually exclusive groups for their status at 12 months after randomisation:

- ‘convicted DV offence’ BOCSAR convicted domestic violence (DV) offence*
- ‘possible DV offence’ Possible conviction, pending court case (from charge report)
- ‘no offence’ No convicted DV offence or pending offences

Time is based upon the date of the offence, not the date of the conviction or charge date. If the participant is taken into custody within this 12 month period, then the 12 month period will be extended to account for this time in custody. Eg. if a participant is in custody for 3 months, then the total time for this participant to have a primary outcome offence will be extended to 15 months.

*The lawparts that define a domestic violence offence are provided in appendix one. The analysis conducted will follow that given in Section [4.6.1](#_bookmark38) [Primary analysis.](#_bookmark38)

- 1. **Subgroup analyses**

We propose to analyse the effect of treatment by treatment adherence. This will be based upon the 12 month average of the self reported question:

“How many days per week, on average, did you actually ingest your study medication?”.

Participants will be categorised into one of the following groups:

- Most adherent patients (≥70% of medications over one year)
- Medium adherent patient (30.0-69.9% of medications over one year)
- Least adherent patients (<30% of medications over one year)
  1. **Exploratory analyses**

Exploratory analyses will look at the following comparator groups:

- Participants that enter run in and are not randomised (RUNIN)
- Randomised to sertraline (ACTIVE)
- Randomised to placebo (CONTROL)

The primary analysis detailed in Section [4.6.1](#_bookmark38) [Primary analysis](#_bookmark38) will compare the effect of treatment between ACTIVE and CONTROL. However, as the primary outcome data will be obtained from an external source to the trial database (BOCSAR), we will have data for run-in patients also. Therefore exploratory analyses to compare CONTROL and RUNIN will enable us to estimate if there were any trial biases introduced, and another analysis to compare RUNIN and ACTIVE will also give insights to the generalisability of the results.

- 1. **Safety analyses**

Safety analyses will be conducted in the safety population, see Section [3.6:](#_bookmark28) [Analysis populations.](#_bookmark28)

- - 1. **Death by suicide**

The deaths will be summarised by treatment, including the causes of death. Death by suicide is of primary interest.

- - 1. **Drug overdose**

Drug overdoses are a particular SAE of interest, and will be summarised by treatment.

- - 1. **Development of serotonin toxicity**

Development of serotonin toxicity (serotonin syndrome) is a particular SAE of interest, and will be summarised by treatment.

- - 1. **Serious adverse events**
       1. ***Definition***

In the case of Serious Adverse Event (SAE), the event is recorded and initially assessed by the Research Nurse or Psychiatrist, prior to consulting with the Clinical Coordinator. Following this, the medical assessment committee (MAC) - responsible for assessing individual medical eligibility, possible drug interactions, side-effects, and monitoring SAEs throughout the trial – will be informed. The MAC includes esteemed medical/psychiatric practitioners who include: Professor Vaughan Carr (Committee Chair), Dr Andrew Ellis, Professor David Greenberg, Professor Alison Jones, Mr Lee Knight (Clinical Coordinator), Professor Peter Schofield, and Professor Kay Wilhelm. Information is provided to the MAC as soon as it comes to light by the Research Nurse and Clinical Coordinator liaising with other health-related services (e.g., Hospital Emergency Departments, Participant’s General Practitioners), so appropriate action can be taken. In the most serious of cases, discontinuation from the trial may be recommended.

- - - 1. ***Summary of SAE***

The total number of SAE, as well as the number of patients with at least one SAE will be summarised by treatment. SAEs will also be summarised by category and whether the event was considered to be related to treatment.

A listing of all SAE will be given in an appendix.

**References**

Gamble C, Krishan A, Stocken D, et al. Guidelines for the Content of Statistical Analysis Plans in Clinical Trials. *JAMA.* 2017;318(23):2337–2343. doi:10.1001/jama.2017.18556

1. **Appendix One**
   1. **Domestic violence lawparts**

Below lists the lawparts that are considered to be domestic violence related.

| 64721 | 64759 | 64798 | 64839 | 64878 | 67773 |
| --- | --- | --- | --- | --- | --- |
| 64722 | 64760 | 64799 | 64840 | 64879 | 67800 |
| 64723 | 64761 | 64800 | 64841 | 64880 | 67801 |
| 64724 | 64762 | 64801 | 64842 | 64881 | 67804 |
| 64725 | 64763 | 64802 | 64843 | 64882 | 67813 |
| 64726 | 64764 | 64803 | 64844 | 64883 | 67831 |
| 64727 | 64765 | 64804 | 64846 | 64884 | 67832 |
| 64728 | 64766 | 64805 | 64847 | 64885 | 67833 |
| 64729 | 64767 | 64806 | 64848 | 64886 | 67834 |
| 64730 | 64768 | 64808 | 64849 | 64887 | 67835 |
| 64731 | 64769 | 64809 | 64850 | 64888 | 67836 |
| 64732 | 64770 | 64810 | 64851 | 64889 | 67837 |
| 64733 | 64771 | 64811 | 64852 | 64890 | 67838 |
| 64734 | 64772 | 64812 | 64853 | 64891 | 69055 |
| 64735 | 64773 | 64813 | 64854 | 64892 | 69120 |
| 64736 | 64774 | 64814 | 64855 | 64893 | 70719 |
| 64737 | 64775 | 64815 | 64856 | 64894 | 70722 |
| 64738 | 64776 | 64816 | 64857 | 64895 | 70724 |
| 64739 | 64777 | 64817 | 64858 | 64896 | 70726 |
| 64740 | 64778 | 64818 | 64859 | 64897 | 70732 |
| 64741 | 64779 | 64819 | 64860 | 64898 | 70753 |
| 64742 | 64780 | 64820 | 64861 | 64899 | 70754 |
| 64743 | 64781 | 64821 | 64862 | 64900 | 73450 |
| 64744 | 64782 | 64822 | 64863 | 64901 | 73453 |
| 64745 | 64783 | 64823 | 64864 | 64902 | 75173 |
| 64746 | 64784 | 64824 | 64865 | 64903 | 75175 |
| 64747 | 64785 | 64825 | 64866 | 64904 | 76943 |
| 64748 | 64786 | 64826 | 64867 | 64905 | 76945 |
| 64749 | 64787 | 64827 | 64868 | 64906 | 76946 |
| 64750 | 64788 | 64828 | 64869 | 64907 | 76948 |
| 64751 | 64789 | 64829 | 64870 | 64908 | 77096 |
| 64752 | 64790 | 64830 | 64871 | 64909 | 77099 |
| 64753 | 64791 | 64831 | 64872 | 65020 | 77100 |
| 64754 | 64793 | 64832 | 64873 | 67764 | 77103 |
| 64755 | 64794 | 64833 | 64874 | 67766 | 77850 |
| 64756 | 64795 | 64835 | 64875 | 67768 | 82221 |
| 64757 | 64796 | 64837 | 64876 | 67769 | 82222 |
| 64758 | 64797 | 64838 | 64877 | 67771 | 85915 |

**Supplementary Results**

| **Supplementary Table 1: Treatment adherence by week and treatment (of all randomised)** | | |
| --- | --- | --- |
| **Characteristic** | **Placebo, N = 311^1^** | **Sertraline, N = 319^1^** |
| Week 0 | 283 (91%) | 296 (93%) |
| Week 2 | 254 (82%) | 256 (80%) |
| Week 6 | 201 (65%) | 204 (64%) |
| Week 10 | 179 (58%) | 186 (58%) |
| Week 14 | 156 (50%) | 171 (54%) |
| Week 18 | 141 (45%) | 160 (50%) |
| Week 22 | 124 (40%) | 139 (44%) |
| Week 26 | 117 (38%) | 131 (41%) |
| Week 30 | 110 (35%) | 117 (37%) |
| Week 34 | 98 (32%) | 114 (36%) |
| Week 38 | 88 (28%) | 103 (32%) |
| Week 42 | 89 (29%) | 101 (32%) |
| Week 46 | 75 (24%) | 83 (26%) |
| Week 50 | 75 (24%) | 84 (26%) |
| Week 54 | 68 (22%) | 67 (21%) |
| ^1^n (%) for adherence defined as proportion of participants taking 70% of more of their medication of all randomised | | |

| **Supplementary Table 2: Serious adverse events and adverse events by treatment arm** | | | | |
| --- | --- | --- | --- | --- |
| **Group** | **Characteristic** | **Active run-in**  **N = 166** | **Placebo**  **N = 311** | **Sertraline**  **N = 319** |
| Total events per person |  |  |  |  |
|  | 0 | 155 (93.4%) | 282 (90.7%) | 297 (93.1%) |
|  | 1 | 9 (5.4%) | 16 (5.1%) | 14 (4.4%) |
|  | 2 | 1 (0.6%) | 8 (2.6%) | 6 (1.9%) |
|  | 3 | 1 (0.6%) | 1 (0.3%) | 1 (0.3%) |
|  | 4 | 0 (0%) | 3 (1.0%) | 0 (0%) |
|  | 6 | 0 (0%) | 1 (0.3%) | 0 (0%) |
|  | 7 | 0 (0%) | 0 (0%) | 1 (0.3%) |

| **Supplementary Table 3: Serious adverse event types by treatment arm** | | | | |
| --- | --- | --- | --- | --- |
|  | **Characteristic** | **Active run-in**  **N = 166** | **Placebo**  **N = 311** | **Sertraline**  **N = 319** |
| Event types | Seizure | 0 (0%) | 1 (0.3%) | 2 (0.6%) |
|  | Burns - Acid | 0 (0%) | 1 (0.3%) | 0 (0%) |
|  | Psychosis | 0 (0%) | 3 (1.0%) | 1 (0.3%) |
|  | Anxiety/Panic/Situational Crisis / Adjustment disorder | 4 (2.4%) | 1 (0.3%) | 2 (0.6%) |
|  | Motor vehicle accident | 0 (0%) | 0 (0%) | 1 (0.3%) |
|  | Delayed ejaculation | 0 (0%) | 6 (1.9%) | 4 (1.3%) |
|  | Depressed mood | 0 (0%) | 2 (0.6%) | 2 (0.6%) |
|  | 2^nd^ degree liquid burn | 0 (0%) | 1 (0.3%) | 0 (0%) |
|  | Infection(s) | 1 (0.6%) | 1 (0.3%) | 1 (0.3%) |
|  | Impulsive overdose (not suicide attempt) | 1 (0.6%) | 2 (0.6%) | 1 (0.3%) |
|  | Pneumothorax | 0 (0%) | 0 (0%) | 1 (0.3%) |
|  | Misadministration of medication | 0 (0%) | 1 (0.3%) | 0 (0%) |
|  | Suicidal ideation | 0 (0%) | 1 (0.3%) | 0 (0%) |
|  | Sleep disturbance | 0 (0%) | 4 (1.3%) | 0 (0%) |
|  | Diagnosis deferred | 0 (0%) | 1 (0.3%) | 0 (0%) |
|  | Poverty of thought | 0 (0%) | 1 (0.3%) | 0 (0%) |
|  | Headache | 1 (0.6%) | 2 (0.6%) | 1 (0.3%) |
|  | Lethargic/Sedation | 0 (0%) | 1 (0.3%) | 1 (0.3%) |
|  | Suppressed appetite | 0 (0%) | 0 (0%) | 1 (0.3%) |
|  | Teeth grinding | 0 (0%) | 1 (0.3%) | 1 (0.3%) |
|  | Gastric disturbance | 0 (0%) | 3 (1.0%) | 1 (0.3%) |
|  | Sore throat | 0 (0%) | 1 (0.3%) | 0 (0%) |
|  | Bell's palsy | 0 (0%) | 0 (0%) | 2 (0.6%) |
|  | Rash | 1 (0.6%) | 0 (0%) | 0 (0%) |
|  | Cramps | 0 (0%) | 1 (0.3%) | 0 (0%) |
|  | Hyperextension injury | 0 (0%) | 1 (0.3%) | 0 (0%) |
|  | Methamphetamine intoxication | 0 (0%) | 1 (0.3%) | 0 (0%) |
|  | Stimulant overdose | 0 (0%) | 1 (0.3%) | 0 (0%) |
|  | Deliberate self-harm | 0 (0%) | 0 (0%) | 1 (0.3%) |
|  | Paranoid ideation | 1 (0.6%) | 1 (0.3%) | 0 (0%) |
|  | Pain in groin area | 0 (0%) | 1 (0.3%) | 0 (0%) |
|  | Non-accidental overdose | 1 (0.6%) | 0 (0%) | 0 (0%) |
|  | Suspected infection | 0 (0%) | 1 (0.3%) | 0 (0%) |
|  | Accidental overdose | 0 (0%) | 1 (0.3%) | 0 (0%) |
|  | Multiple physical health issues | 0 (0%) | 1 (0.3%) | 0 (0%) |
|  | ETOH intoxication | 0 (0%) | 1 (0.3%) | 0 (0%) |
|  | Fracture | 0 (0%) | 3 (1.0%) | 1 (0.3%) |
|  | Back injury | 0 (0%) | 0 (0%) | 1 (0.3%) |
|  | Hyperglycaemia | 1 (0.6%) | 0 (0%) | 0 (0%) |
|  | Shakiness | 1 (0.6%) | 0 (0%) | 0 (0%) |
|  | Acute hepatitis | 1 (0.6%) | 0 (0%) | 0 (0%) |
|  | Viral illness | 0 (0%) | 0 (0%) | 1 (0.3%) |
|  | Death** | 0 (0%) | 1 (0.3%) | 0 (0%) |
|  | Minor overdose | 1 (0.6%) | 0 (0%) | 1 (0.3%) |
|  | Alcoholic cirrhosis | 0 (0%) | 1 (0.3%) | 0 (0%) |
|  | Swollen ankles | 0 (0%) | 0 (0%) | 1 (0.3%) |
|  | Surgery | 0 (0%) | 1 (0.3%) | 2 (0.6%) |
|  | Workplace injury | 0 (0%) | 0 (0%) | 1 (0.3%) |
|  | Accidental laceration | 0 (0%) | 1 (0.3%) | 0 (0%) |
|  | Dehydration | 0 (0%) | 1 (0.3%) | 0 (0%) |

Multiple events within a single patient are only counted once.

** One death occurred in a participant engaged in the trial which we were aware of, and one further death was identified from the data-linkage which we were unaware of due to him being uncontactable (cause – cardiovascular disease).

| **Supplementary Table 4. Offending Outcomes in the Second Year (Months 13-24) Stratified by Trial Engagement Among Participants Event-Free at 12 Months** | | | | |
| --- | --- | --- | --- | --- |
| **Outcome** | **Subgroup** | **Group** | **Events / N (%)** | **RR (95% CI)** |
| General violent offending | Engaged Beyond 12 Months^†^ | Placebo | 8 / 62 (12.9%) | 1.36 (0.60, 3.07) |
|  |  | Sertraline | 13 / 74 (17.6%) |  |
|  | Disengaged Before 12 Months^‡^ | Placebo | 26 / 171 (15.2%) | 1.03 (0.62, 1.70) |
|  |  | Sertraline | 25 / 160 (15.6%) |  |
| DV offending | Engaged Beyond 12 Months^†^ | Placebo | 9 / 65 (13.8%) | 0.56 (0.21, 1.50) |
|  |  | Sertraline | 6 / 77 (7.8%) |  |
|  | Disengaged Before 12 Months^‡^ | Placebo | 24 / 161 (14.9%) | 0.92 (0.54, 1.56) |
|  |  | Sertraline | 23 / 168 (13.7%) |  |

RR = Relative Risk; CI = Confidence Interval; DV = Domestic Violence.

† "Engaged Beyond 12 Months" subgroup includes participants who were event-free at 12 months and had their last study contact after Week 50.

‡ "Disengaged Before 12 Months" subgroup includes participants who were event-free at 12 months and had their last study contact at or before Week 50.

| **Supplementary Table 5. Violent and Domestic Violent Offending at 12 and 24 months by Adherence** | | | | |
| --- | --- | --- | --- | --- |
| **Outcome** | **Most/Medium Adherent**  **Events / Total (%)** | **RR (95% CI)** | **Least Adherent**  **Events/Total (%)** | **RR (95% CI)** |
| **Violent Offending** |  |  |  |  |
| **12 Months** |  |  |  |  |
| Placebo | 34/170 (20.0%) | 1.00 (ref) | 36/141 (25.5%) | 1.00 (ref) |
| Sertraline | 42/187 (22.5%) | 1.12 (0.75, 1.69) | 30/132 (22.7%) | 0.89 (0.58, 1.36) |
| **24 Months** |  |  |  |  |
| Placebo | 50/170 (29.4%) | 1.00 (ref) | 54/141 (38.3%) | 1.00 (ref) |
| Sertraline | 66/187 (35.3%) | 1.20 (0.89, 1.63) | 44/132 (33.3%) | 0.87 (0.63, 1.20) |
| **Domestic Violence Offending** |  |  |  |  |
| **12 Months** |  |  |  |  |
| Placebo | 38/170 (22.4%) | 1.00 (ref) | 39/141 (27.7%) | 1.00 (ref) |
| Sertraline | 30/187 (16.0%) | 0.72 (0.46, 1.10) | 31/132 (23.5%) | 0.85 (0.56, 1.27) |
| **24 Months** |  |  |  |  |
| Placebo | 56/170 (32.9%) | 1.00 (ref) | 55/141 (39.0%) | 1.00 (ref) |
| Sertraline | 43/187 (23.0%) | **0.70 (0.49, 0.98)*** | 47/132 (35.6%) | 0.91 (0.67, 1.24) |

Note: RR = Risk Ratio; CI = Confidence Interval; * p<0.05

Adherence categories were derived based on weekly self-reports of medication ingestion during attended visits at scheduled timepoints. For each participant, the cumulative number of attended visits where adherence was reported was calculated based on their status up to their final recorded visit at 12 months post-randomisation. This count was then expressed as a percentage of the total number of expected visits planned over the first year (fixed at 15 for all participants).

Participants were classified based on this final percentage as: Most adherent (≥70% of total potential visits), Medium adherent (30.0–69.9% of total potential visits), and Least adherent (<30% of total potential visits).

For the analysis presented in this table, the Most adherent and Medium adherent groups were combined into a single 'Most/Medium Adherent' category (representing adherence confirmed at ≥30% of the total potential visits) to increase analytic power.

| **Supplementary Table 6. Baseline characteristics of the non-randomized (run-in) and randomized participants** | | | |
| --- | --- | --- | --- |
| **Characteristics** | **Non-Randomized**  **N = 166** | **Randomized**  **n = 630** | **P value** |
| Age at randomisation (years), Median (IQR) | 29 (23, 37) | 32 (25, 40) | 0.15 |
| Unknown | 2 | 9 |  |
| Country of Birth |  |  | 0.68 |
| Australia | 132 (82%) | 520 (83%) |  |
| Other | 30 (19%) | 107 (17%) |  |
| Unknown | 4 | 3 |  |
| Indigenous status |  |  | 0.42 |
| Indigenous | 53 (32%) | 188 (30%) |  |
| Non-Indigenous | 111 (68%) | 440 (70%) |  |
| Unknown | 2 | 2 |  |
| Marital status |  |  | 0.54 |
| Single | 92 (56%) | 328 (52%) |  |
| Partner | 39 (24%) | 154 (25%) |  |
| Married/Defacto | 22 (14%) | 99 (16%) |  |
| Separated | 5 (3%) | 34 (5.4%) |  |
| Divorced | 5 (3%) | 10 (1.6%) |  |
| Widowed | 0 (0) | 1 (0.2%) |  |
| Unknown | 3 | 4 |  |
| Number of children/stepchildren | 1 (0, 3) | 1 (0, 3) | 0.25 |
| Unknown | 3 | 2 |  |
| Accommodation |  |  | 0.88 |
| Renting | 108 (66%) | 425 (68%) |  |
| Home/Family | 38 (23%) | 136 (22%) |  |
| Unsettled | 7 (4%) | 30 (4.8%) |  |
| Sleeping rough | 3 (2%) | 7 (1.1%) |  |
| Other | 6 (4%) | 30 (4.8%) |  |
| Unknown | 4 | 2 |  |
| Highest education level |  |  | 0.10 |
| Never attended school | 4 (2%) | 1 (0.2%) |  |
| Primary school | 3 (2%) | 14 (2.2%) |  |
| Left school with no qualification | 48 (29%) | 200 (32%) |  |
| School certificate | 62 (38%) | 221 (35%) |  |
| HSC/Leaving certificate | 22 (14%) | 63 (10%) |  |
| College/Diploma | 6 (4%) | 25 (4%) |  |
| Technical or Trade qualification | 15 (9%) | 87 (14%) |  |
| Degree/Tertiary qualification | 3 (2%) | 13 (2%) |  |
| Unknown | 3 | 6 |  |
| Working in the past 6 months | 77 (47%) | 380 (61%) | <0.01 |
| Unknown | 4 | 5 |  |
| Juvenile detention | 57 (35%) | 140 (22%) | <0.01 |
| Unknown | 4 | 5 |  |
| Parent in prison when child | 37 (23%) | 139 (22%) | 0.78 |
| Unknown | 7 | 5 |  |
| Number of previous offenses*** |  |  |  |
| Any offense | 12 (7, 22) | 9 (4, 15) | <0.001 |
| Violent offense | 2 (1, 4) | 2 (1, 4) | 0.68 |
| Domestic violence (DV) offense | 2 (0, 4) | 2 (0, 4) | 0.34 |
| Violent DV offenses | 1 (0, 2) | 1 (0, 2) | 0.36 |
| Barratt Impulsiveness Scale - Total Score, Median (IQR) ^‡^ | 85 (77, 93) | 85 (77, 92) | 0.59 |
| Kessler-10 - Psychological Distress Scale, Median (IQR) ^§^ | 15 (9, 21) | 14 (8, 21) | 0.67 |
| SF-12 - Mental Health Score, Median (IQR) ^¶^ | 43 (30, 51) | 43 (30, 52) | 0.23 |
| SF-12 - Physical Health Score, Median (IQR) ^¶^ | 55 (51, 58) | 55 (51, 58) | 0.78 |
| AIAQ Total Score, Median (IQR) ^‖^ | 76 (62, 93) | 72 (56, 88) | 0.03 |
| STAXI - Anger Expression Index, Median (IQR) ** | 54 (42, 63) | 51 (43, 60) | 0.26 |
| EYSENCK – Impulsivity Score, Median (IQR) ^††^ | 14 (11, 16) | 14 (11, 16) | 0.98 |
| Beck Depression Inventory Score, Median (IQR) ^‡‡^ | 10 (5, 16) | 9 (4, 15) |  |

Percentages may not total 100 due to rounding. IQR denotes interquartile range.

‡ Barratt Impulsiveness Scale: Higher scores indicate greater impulsivity.

§ Kessler-10 Psychological Distress Scale: Higher scores indicate greater psychological distress.

¶ SF-12 Mental and Physical Health components: Higher scores indicate better health status.

‖ AIAQ (Anger, Irritability and Assault Questionnaire) Total Score: Higher scores indicate greater anger and irritability.

** STAXI (State-Trait Anger Expression Inventory) Anger Expression Index: Higher scores indicate greater anger expression.

†† EYSENCK Impulsivity Score: Higher scores indicate greater impulsivity.

‡‡ Beck Depression Inventory: Higher scores indicate more severe depressive symptoms.

***Within five years prior to randomization

| **Supplementary Table 7. Multivariable Adjusted Analysis of Violent and Domestic Violence Offending at 12 and 24 Months: Randomized Groups vs. Non-Randomized Run-in Group** | | | | | | | | |
| --- | --- | --- | --- | --- | --- | --- | --- | --- |
|  | **Violent offending** | | | | **Domestic Violence offending** | | | |
| **Characteristics** | **12 months** | | **24 months** | | **12 months** | | **24 months** | |
|  | **Adjusted RR (95% CI)** | **P value** | **Adjusted RR (95% CI)** | **P value** | **Adjusted RR (95% CI)** | **P value** | **Adjusted RR (95% CI)** | **P value** |
| **Group** |  |  |  |  |  |  |  |  |
| Placebo | 0.75 (0.55, 1.02) | 0.064 | 0.78 (0.62, 0.98) | 0.032* | 0.74 (0.55, 1.00) | 0.049* | 0.85 (0.67, 1.08) | 0.187 |
| Sertraline | 0.73 (0.54, 0.99) | 0.047* | 0.80 (0.64, 1.00) | 0.053 | 0.55 (0.39, 0.75) | <0.001*** | 0.66 (0.51, 0.85) | 0.002** |
| Run-in | 1.00 |  | 1.00 |  | 1.00 |  | 1.00 |  |
| **Employment (past 6 months)** |  |  |  |  |  |  |  |  |
| Employed | 1.01 (0.79, 1.30) | 0.945 | 1.01 (0.84, 1.22) | 0.906 | 0.98 (0.76, 1.26) | 0.868 | 0.98 (0.80, 1.20) | 0.862 |
| Not employed | 1.00 |  | 1.00 |  | 1.00 |  | 1.00 |  |
| **Juvenile Detention** |  |  |  |  |  |  |  |  |
| Detained | 1.31 (1.01, 1.69) | 0.045* | 1.34 (1.11, 1.63) | 0.002** | 0.97 (0.73, 1.30) | 0.847 | 1.09 (0.87, 1.35) | 0.468 |
| Not detained | 1.00 |  | 1.00 |  | 1.00 |  | 1.00 |  |
| **Prior Offense (within 5 Years prior to randomisation)** |  |  |  |  |  |  |  |  |
| Yes | 1.27 (0.37, 4.35) | 0.709 | 1.24 (0.49, 3.15) | 0.655 | 1.26 (0.37, 4.36) | 0.713 | 1.84 (0.52, 6.44) | 0.344 |
| No | 1.00 |  | 1.00 |  | 1.00 |  | 1.00 |  |
| **AIAQ total score** | 1.01 (1.00, 1.02) | 0.001** | 1.01 (1.00, 1.01) | <0.001*** | 1.01 (1.00, 1.01) | 0.122 | 1.01 (1.00, 1.01) | 0.014* |

1. Men who do not complete the randomisation visit (eg. abscond prior to receiving study medication) will not be included in the intention to treat population [↑](#footnote-ref-1)
2. The BOCSAR ROD database was requested on 24^th^ January 2025 for the final analysis. [↑](#footnote-ref-2)
3. Adjudication of outcomes for participants with less than two years of database follow-up for “possible offences” was rendered unnecessary as, at the time of final data linkage, all participants had accrued at least two years of follow-up data within the Reoffending Database. [↑](#footnote-ref-3)
